# Delta power surge and alpha power decline in traumatic brain injury recovery: A quantitative EEG analysis of the CAPTAIN-rTMS trial

**DOI:** 10.1017/cts.2025.10159

**Published:** 2025-09-25

**Authors:** Livia Livinț-Popa, Vlad-Florin Chelaru, Diana Chertic-Dăbală, Diana Chira, Olivia Verișezan-Roșu, Victor Dăbală, Nicu Drăghici, Enola Maer, Ştefan Strilciuc, Dafin Mureșanu

**Affiliations:** 1 Department of Neurosciences, Iuliu Haţieganu University of Medicine and Pharmacy, Cluj-Napoca, Romania; 2 RoNeuro Institute for Neurological Research and Diagnostic, Cluj-Napoca, Romania; 3 IMOGEN Institute, Centre of Advanced Research Studies, Cluj-Napoca, Romania; 4 Research Center for Functional Genomics, Biomedicine and Translational Medicine, Iuliu Hatieganu University of Medicine and Pharmacy, Cluj-Napoca, Romania

**Keywords:** TBI, rTMS, qEEG, cerebrolysin, power spectral density

## Abstract

**Introduction:**

Traumatic brain injury (TBI) is a leading cause of disability and death. Both repetitive transcranial magnetic stimulation (rTMS) and Cerebrolysin (CRB) are promising therapies regulating neural plasticity. This study aimed to assess the changes in resting-state brain activity following CRB, rTMS, or combined CRB-rTMS therapy.

**Methods:**

This secondary analysis of the CAPTAIN-rTMS trial analyzed eyes-closed segments from EEG recordings at 30 days (baseline) and 180 days (after treatment) respectively. We computed relative power spectral densities for delta, theta, alpha and beta frequency bands, for the entire scalp and different regions. We conducted neuropsychological assessments and evaluated the correlations between resting-state relative power spectral density values and neuropsychological assessment performance.

**Results:**

We analyzed a total of 50 patients. For the entire scalp, we found statistically significant decreases in relative alpha power (*p* = 0.02) and significant increases in relative delta power (*p* = 0.02), further subgroup analysis showing differences between visits in the CRB + sham group (paired Cliff’s *δ* = 0.6, *p* = 0.012 for Delta band, *δ* = 0.6, *p* = 0.064 for Alpha band). The differences were higher in the central (alpha *p* = 0.004, delta *p* = 0.002) and parietal (alpha *p* = 0.012, delta *p* = 0.03), and lower in the frontal (alpha *p* = 0.05, delta *p* = 0.026), temporal (alpha *p* = 0.065, delta *p* = 0.077), and occipital (alpha *p* = 0.064, delta *p* = 0.084) regions. Neuropsychological tests performance was negatively correlated with resting-state relative delta power, and positively correlated with alpha power.

**Conclusion:**

We found overall slowing of brain electrical activity during recovery after TBI, which was further influenced by rTMS and CRB treatment. Resting-state relative power spectral densities correlate with neuropsychological measurements.

## Introduction

Traumatic brain injury (TBI) represents a nonhomogeneous clinical syndrome that causes lasting disability and death worldwide, as well as considerable medical expenditures. With an estimated prevalence of 50 million individuals annually, TBI contributes most to long-term neurological disability [1]. It is broadly defined as an alteration in the normal physiology of the brain as a consequence of an external physical force, producing various neurobehavioral sequelae such as post-traumatic epilepsy, chronic pain, cognitive and motor impairments, sleep, mood, and memory disorders or modifications in the level of consciousness spanning over different temporal scales [2–5]. Both individual and societal factors emphasize the need for better interventions and strategies to increase post-trauma integration.

After the acute events following the disruption of brain tissue subside, a dynamic period of functional and anatomical neural network reorganization begins characterized by activity-dependent synaptic strength changes, decreases in the amount of GABA-ergic inhibition, neurogenesis, and axonal regeneration [6, 7]. Natural developments in plasticity that promote recovery following a TBI may result in maladaptive outcomes if proper therapeutic interventions and guidance are not provided in a timely manner.

Several pharmacological and neuromodulation strategies (alone or in combination) that modulate the process of neuroplasticity are very effective in the setting of complex brain injuries. Repetitive transcranial magnetic stimulation (rTMS) represents a noninvasive instrument that uses electromagnetic induction to generate electrical currents in a restricted area of the superficial cortex, leading to neural tissue depolarization and local information processing alteration [8]. Although the immediate effects on cortical excitability depend on the parameters used, rTMS effects outlast the stimulation period by activating the mechanisms of long-term potentiation and depression at the synaptic level [9–11]. The therapeutic potential of rTMS has been proven in numerous neurological and psychiatric clinical areas such as depression, neuropathic pain, fibromyalgia, in the acute stage of stroke for hand motor impairments, Parkinson’s disease, and post-traumatic stress disorder [8, 9]. In the context of TBI research, the application of rTMS demonstrated the most significant advantages in addressing cognitive disturbances (such as attention and memory), mood disorders, and sensorimotor learning [6].

Repetitive transcranial magnetic stimulation protocols can be used in conjunction with pharmacological agents with a precise mechanism of action to investigate the physiological processes that regulate plasticity and cortical excitability [12]. Alternatively, neurostimulation can also be used as an adjunct to pharmacological interventions to target various refractory symptoms and accelerate clinical recovery. Cerebrolysin (CRB) emerges as a promising therapeutic agent to mitigate secondary injuries following TBI, fostering the physiological processes of neuronal repair and neuroprotection replicating the effects of endogenous neurotrophic factors. Its composition resides in a mixture of peptides and low-molecular-weight amino acids obtained from purified porcine brain proteins [13–15]. Several clinical trials assessing the impact of CRB in the neurorehabilitation of patients with moderate–severe TBI demonstrated its superiority compared to placebo on a comprehensive battery of outcome scales [14, 16].

By integrating TMS protocols with brain imaging techniques, it is possible to objectively assess how focal brain stimulation affects local and remote intrinsic brain activity [17]. Building upon the results presented by Verisezan-Rosu and colleagues [18] and our previous secondary analysis regarding frequency band ratios [19], our aim for this secondary analysis was to explore the potential changes in resting-state brain activity (as evaluated through relative power spectral density values) in TBI patients treated with CRB, rTMS, or combined CRB-rTMS therapy, as well as to examine the correlation between relative power spectral density values and neuropsychometric scores and performance.

## Materials and methods

This was an exploratory secondary analysis of the CAPTAIN-rTMS three-arm, double-blind, phase II randomized controlled trial [18]. Briefly, between April 2018 and September 2021, we recruited patients with moderate-severe TBI, with ages between 18 and 80 years of age, with TBI onset in last 30 days before screening; TBI was confirmed by focal or diffuse lesions on CT or MRI; moreover, in order to be included, patients were required to have a Pre-Trauma Karnofsky Index of 100 and be willing to adhere to the study protocol. Exclusion criteria for the original study were metallic implants or medical implanted devices, past intracranial surgery, penetrating brain injuries, major comorbidities (such as cancer, heart or coronary disease, diabetes, addictions, psychiatric disorders, epilepsy, degenerative or inflammatory pathologies of the nervous system) including chronic medication, conditions that could influence outcome measurements (including signs of intoxication or injury of dominant hand), as well as, for females, pregnancy, lactation, or lack of use of adequate contraceptives. Study visits and treatment administration were conducted at the RoNeuro Institute for Neurological Research and Diagnostic, Cluj-Napoca, Romania, in an outpatient setting. Complete inclusion and exclusion criteria were reported in the original study [18]; additionally, for this analysis, patients were included only if EEG recordings at baseline and last visit were available.

The study protocol was approved by the Institutional Review Board of Iuliu Haţieganu University of Medicine and Pharmacy Cluj-Napoca, Romania (2/08.01.2018), with subsequent amendments (133/15.03/2018 and 118/23.04.2019); the study followed the ethical guidelines of the Helsinki Declaration. Results of this study were reported in accordance with the STROBE (Strengthening the Reporting of Observational Studies in Epidemiology) guidelines, available as Supplementary File S1 [20].

**Figure 1.**
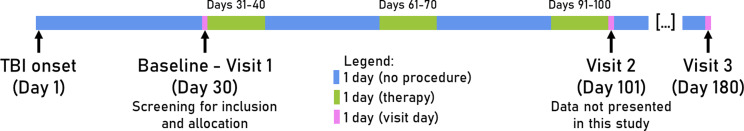
Timeline of the experimental procedure. TBI = traumatic brain injury; rTMS = repetitive transcranial magnetic stimulation.

Patients were randomly assigned, in equal proportions, to one of three treatment regiments: Cerebrolysin and rTMS (CRB + rTMS), Cerebrolysin and sham rTMS (CRB + sham), or placebo and sham rTMS (PLC + sham). Additionally, all patients received standard care after TBI. Study visits included in this secondary analysis were the screening and baseline evaluation considered on day 30 after TBI, as well as the last visit on day 180 after TBI (considered Visit 2 in the original study). Patients received CRB infusions of 30 mL diluted in 0.9% saline solution (250 mL total infusion volume), on days 31–40, 61–70 and 91–100, or placebo infusions amounting to 250 mL of 0.9% saline solution with the same schedule (Figure 1). Repetitive transcranial magnetic stimulation and its sham counterpart were executed on the same schedule as CRB/placebo infusions, using a MagPro X100 (MagVenture, Farum, Denmark) medical device with a figure-8 coil (MCF-B65) for the rTMS-allocated patients or a sham-coil (MCF-P-B65) which mimicked the shape, noise level and cutaneous discomfort as the real counterpart. During the first treatment session, the resting motor threshold was determined as the minimal intensity eliciting motor-evoked potentials of at least 50 µV in the abductor pollicis brevis in five out of ten trials. Then, the rTMS procedure consisted of left dorsolateral prefrontal cortex (DLPFC) stimulation for all patients (irrespective of lesion site) with 10 Hz stimuli, with a total of 1200 stimuli per day and an intensity of 120% of the resting motor threshold (40 trains of 3-second stimulation separated by 20-second inter-train intervals, for a total duration of 33 minutes). Personnel involved in infusion administration or rTMS procedures were not involved in any other activities related to the study, nor were they allowed to disclose information about the treatment procedure.

Participants underwent a battery of neuropsychological assessments to evaluate various aspects of cognitive and executive function, mood, attention, working memory, and associative learning. The evaluation included Montreal Cognitive Assessment (MoCA) [21], Digit Span and Processing Speed Index (PSI) subsets of the Wechsler Adult Intelligence Scale III [22, 23], Stroop Color-Word Test [24], Trail Making Test [25], Hamilton Anxiety Rating Scale [26], Hamilton Rating Scale for Depression [27] and three subsets of the Cambridge Neuropsychological Test Automated Battery (CANTAB): Reaction Time (RTI), Multitasking Test and One Touch Stockings of Cambridge (OTS) [28]. To maintain consistency and eliminate potential bias, all assessments were conducted by the same specialist who was unaware of the participants’ assignment group during both evaluation visits.

A 5-minute eyes-closed (EC) EEG recording was conducted in each session. Participants were instructed to maintain a relaxed, wakeful state without engaging in any specific task during the recording. EEG data was collected using a 32-electrode montage (Easycap, Herrsching, Germany) positioned following the 10–20 International system, including Fp1, Fp2, F3, F4, F7, F8, Fz, C3, C4, Cz, P3, P4, P7, P8, Pz, O1, O2, Oz, FC1, FC2, FC5, FC6, CP1, CP2, CP5, CP6, T7, T8, TP9, TP10, PO9 and PO10. Data was sampled at a rate of 1024 Hz, with a band-pass filter set between 0.5–40 Hz. Before the recording session, the conductance of electrodes was reviewed to maintain impedances below 5 kΩ.

EEG recording de-artifaction was performed by an experienced neurologist using BrainVision V2.1 (Brain Products GmbH, Munich, Germany). Data was downsampled to 512 Hz and re-referenced to the average electrode, and a 50 Hz notch filter was applied to remove power line noise. Independent Component Analysis (based on Infomax algorithm) was used to identify the effect of artifacts such as eye movements, muscle twitching, and heartbeat on the recorded signal; components represented by artifacts were manually removed and the electrode signals were reconstructed. Recording segments containing remaining artifacts were manually rejected. Before data export, de-artifacted segments were compared with the original signal, to ensure that electrical activity specific to the brain was not altered.

We then used Brainstorm version 12.03.2025 [29], a toolbox based on the Matlab framework (The Mathworks Inc., Natick, MA) to generate absolute power spectral density values. We generated Power Spectrum Density (PSD) values using the Welch method with default parameters (window length of one second, window overlap ratio of 50%, physical units. The power spectrum was defined across distinct frequency bands as follows: Delta 0–4 Hz, Theta 4–8 Hz, Alpha 8–13 Hz, and Beta 13–30 Hz. Relative power spectral density values were calculated by dividing the power value for each electrode by the sum of powers in the included frequency spectrum (0–30 Hz). Then, relative PSD values for each scalp zone were computed by averaging the relative PSD values of their respective electrodes, for each frequency separately.

For the statistical analysis, we used R version 4.4.2 [30] with RStudio [31], with the following libraries: R.matlab version 3.7.0 [32] to import power spectral density values from the Brainstorm database, openxlsx version 4.2.8 [33] and stringr version 1.5.1 [34] to interact with spreadsheet files, ggplot2 version 3.5.1 [35], patchwork version 1.3.0 [36] and ggforce version 0.4.2 [37] to create the graphs, as well as MANOVA.RM version 0.5.4 [38] to execute mixed-models, resampling-based ANOVA analysis.

The effects of treatment groups (between-subject variable), visits (within-subject variable) and their interaction on values of relative power spectral density, for each band and scalp zone, were reported using the resampling-based results of the MANOVA.RM analysis. The analysis was executed with 10,000 iterations (the default value), without parallelization, with the seed 20000523 and reporting the first three decimal places of p-values. Furthermore, relative power spectral density values were compared within visits, between groups, using Kruskal–Wallis and Wilcoxon rank sum tests, and Cliff’s δ [39] was used as effect size because of its lack of assumptions regarding the distribution of the data. Power spectral density values were also compared within groups, between visits, using Wilcoxon signed-rank test, and a paired equivalent of Cliff’s δ was used as effect size. The formulae of Cliff’s δ and its paired counterpart are:


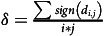
 , 
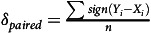


where *X* and *Y* represent the values of the compared groups, subscript *i* and *j* represent the i-th and j-th elements of *X* and *Y* respectively, *n*_*X*_ and *n*_*Y*_ represent the total number of elements in *X* and *Y*, and *d* is the difference matrix such that *d*_*i,j*_ *=Y*_*j*_ *-X*_*i*_.

Correlations between power spectral density values and clinical scale results were computed using Spearman correlation coefficient and its associated test, with p-values being adjusted for multiple testing using the False Discovery Rate method. In case of missing values for neuropsychological assessment, only the value pairs with missing data were excluded.

All analyses were conducted using a significance level of alpha = 0.05. Cliff’s δ values were interpreted according to the following rules: *δ* ≈ 0.2 is a small effect size, *δ* ≈ 0.5 is a medium effect size, and *δ* ≈ 0.8 is a large effect size.

## Results

We analyzed a subgroup of 50 patients from the original study, representing all three treatment groups: CRB + rTMS (*N* = 16), CRB + sham (*N* = 15) and PLC + sham (*N* = 19). Age and gender distributions across groups are available in Table 1.

**Table 1.** Age and gender distributions for the subjects analyzed

	CRB + rTMS	CRB + sham	PLC + sham	p-value
Subjects	16	15	19	
Age (years)				
Mean ± standard deviation	45.313 ± 15.387	53.267 ± 12.115	50.474 ± 17.170	0.39 (Kruskal–Wallis test)
Median (quantile 1 – quantile 3)	42 (35.5 – 57)	52 (44.5 – 60.5)	50 (37 – 63)	
Minimum–maximum	21 – 69	36 – 78	22 – 75	
Females (%)	2 (12.5%)	1 (6.67%)	5 (26.32%)	0.308 (Fisher’s exact test)

CRB = Cerebrolysin; rTMS = repetitive transcranial magnetic stimulation; PLC = placebo; sham refers to a sham procedure of rTMS.

### Power spectral density differences by region

**Figure 2.**
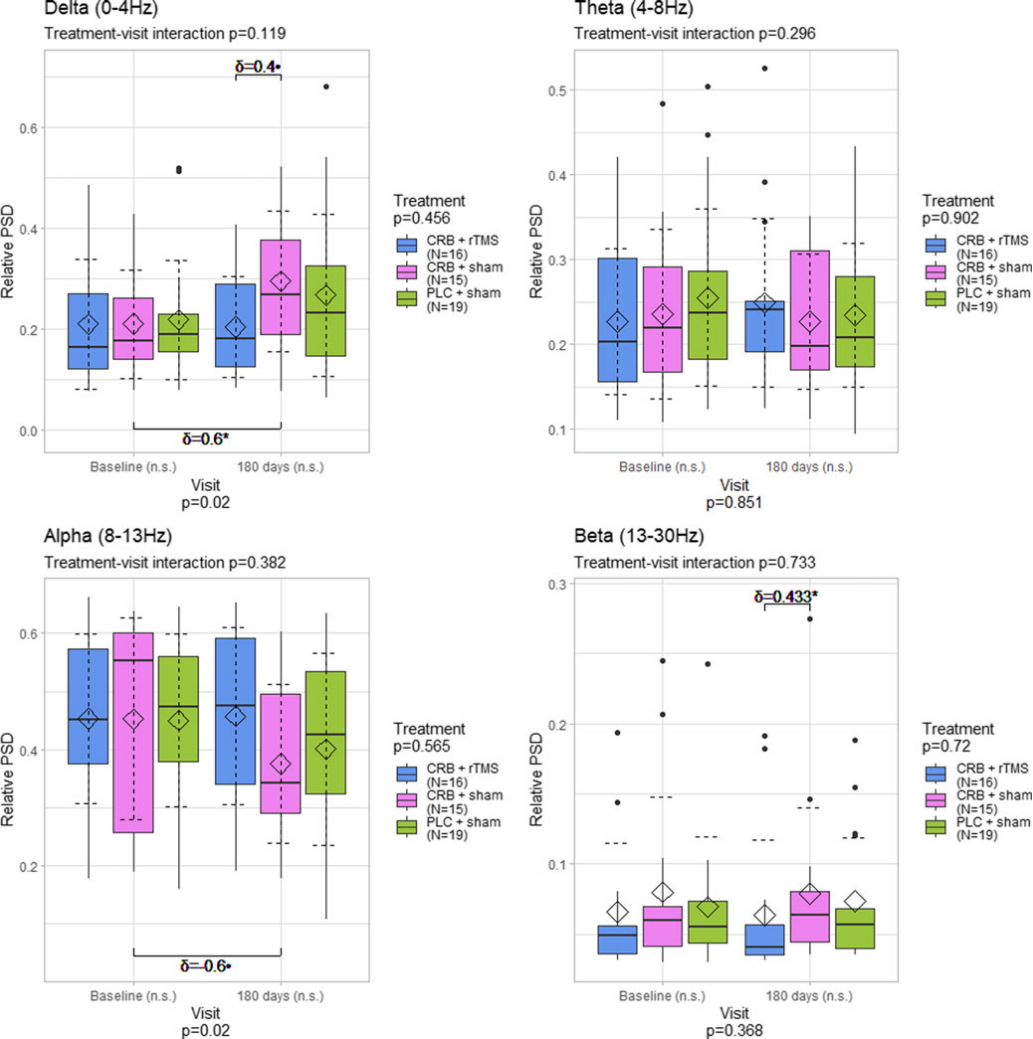
Distribution of power spectral density values averaged for the entire scalp, by frequency band, visit, and treatment group. The diamond represents the mean, and the dashed error lines represent the standard deviation relative to the mean. Resampling-based mixed-models ANOVA p-values specified for each factor; Kruskal–Wallis test was used for comparisons between all groups for each visit; Wilcoxon rank sum test and Cliff’s δ were used for comparisons between groups (upper part of the graphs); Wilcoxon signed rank test and paired Cliff’s δ were used for comparisons between visits (lower part of the graphs); Cliff’s δ values shown for *p* < 0.1. CRB = cerebrolysin; rTMS = repetitive transcranial magnetic stimulation; PLC = placebo; n.s. = not significant; • - *p* < 0.1: * - *p* < 0.05; ** - *p* < 0.01; *** - *p* < 0.001.

Regarding the electroencephalographic activity averaged over the entire cortex, we found statistically significant differences between visits in the alpha (*p* = 0.02) and delta (*p* = 0.02) bands, but not in theta (*p* = 0.851) and beta (*p* = 0.368) bands (Figure 2). Further analysis showed a slight, but not statistically significant, decrease in alpha-band power for CRB + sham group, but not in the CRB + rTMS or PLC + sham groups. We also found a statistically significant increase of medium size in the delta power band in patients treated with CRB and sham rTMS (*p* = 0.012, *δ* = 0.6). There were no significant differences in the theta and beta power bands.

**Figure 3.**
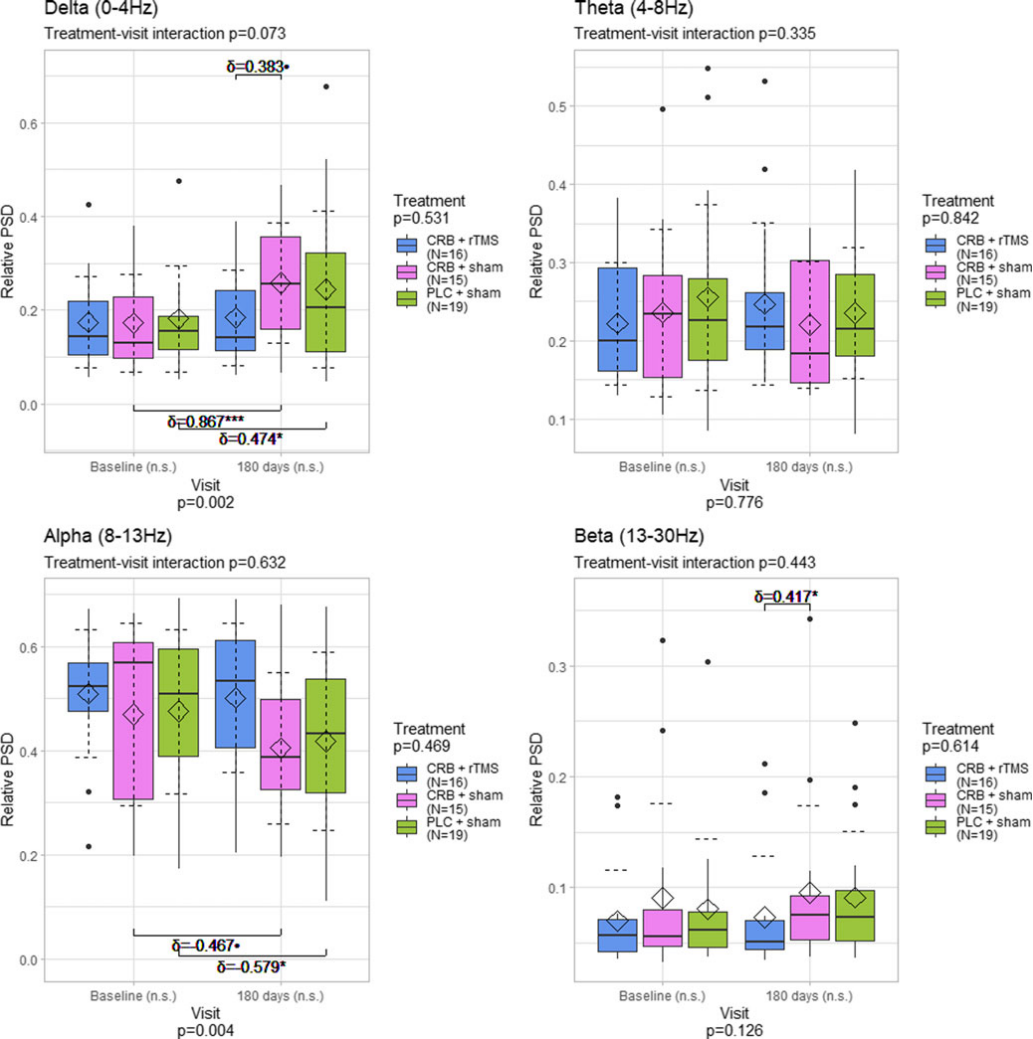
Distribution of power spectral density values averaged for the central electrodes, by frequency band, visit, and treatment group. The diamond represents the mean, and the dashed error lines represent the standard deviation relative to the mean. Resampling-based mixed-models ANOVA p-values specified for each factor; Kruskal–Wallis test was used for comparisons between all groups for each visit; Wilcoxon rank-sum test and Cliff’s δ were used for comparisons between groups (upper part of the graphs); Wilcoxon signed rank test and paired Cliff’s δ were used for comparisons between visits (lower part of the graphs); Cliff’s δ values shown for *p* < 0.1. CRB = cerebrolysin; rTMS = repetitive transcranial magnetic stimulation; PLC = placebo; n.s. = not significant; • - *p* < 0.1: * - *p* < 0.05; ** - *p* < 0.01; *** - *p* < 0.001.

In the central region, we observed the same changes in delta (*p* = 0.002) and alpha (*p* = 0.004) power spectral densities across visits, with further analysis showing significant delta power increases for the groups treated with CRB and sham magnetic stimulation (*p* < 0.001, large difference of *δ* = 0.867) and with placebo and sham magnetic stimulation (*p* = 0.045, medium effect of *δ* = 0.474) respectively. In the alpha band, a statistically significant decrease of medium size was detected in the group treated with placebo and sham stimulation (*p* = 0.045, *δ* = 0.579). There were no significant differences between visits in the theta and beta power bands, but there was a significant difference in the beta band at the last visit between the group treated with CRB and rTMS compared to the group treated with CRB alone (*p* = 0.049, *δ* = 0.417) (Figure 3).

**Figure 4.**
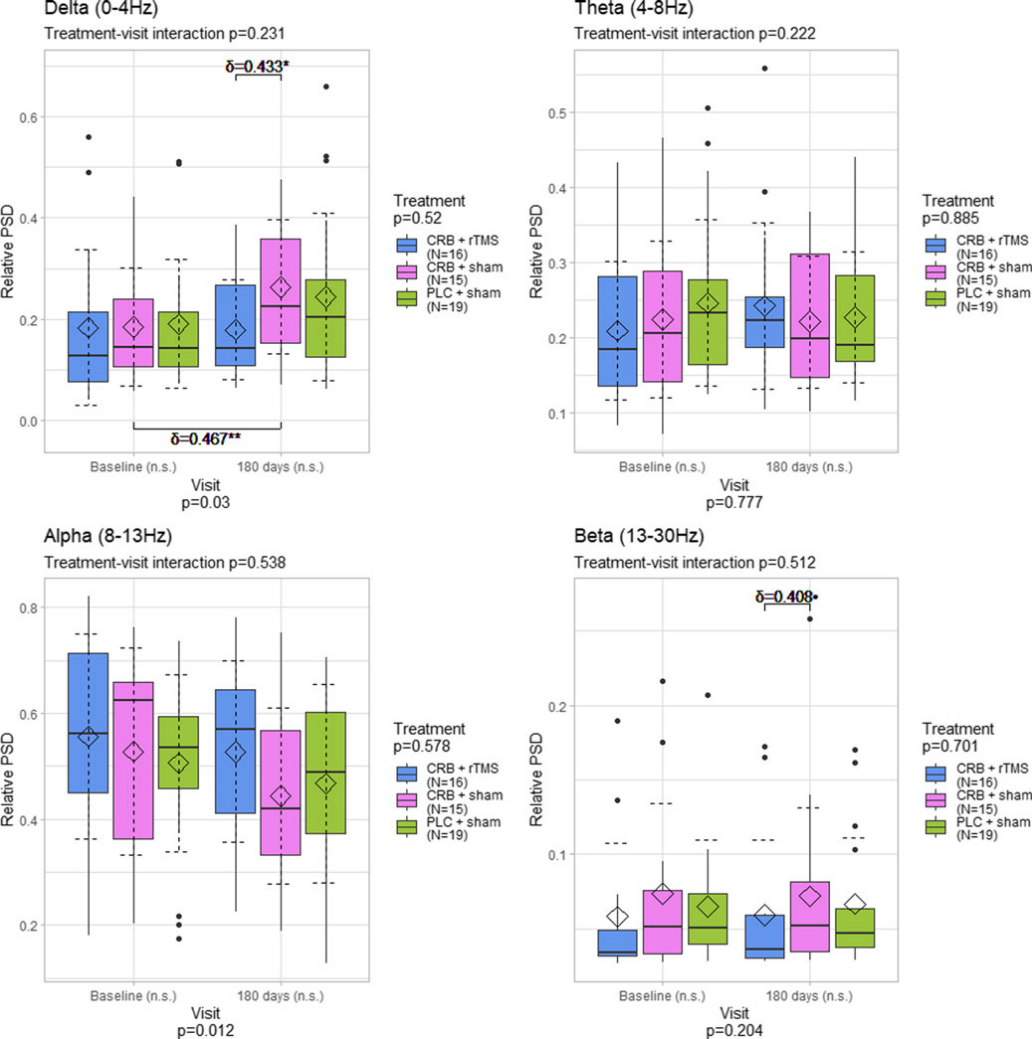
Distribution of power spectral density values averaged for the parietal electrodes, by frequency band, visit, and treatment group. The diamond represents the mean, and the dashed error lines represent the standard deviation relative to the mean. Resampling-based mixed-models ANOVA p-values specified for each factor; Kruskal–Wallis test was used for comparisons between all groups for each visit; Wilcoxon rank-sum test and Cliff’s δ were used for comparisons between groups (upper part of the graphs); Wilcoxon signed rank test and paired Cliff’s δ were used for comparisons between visits (lower part of the graphs); Cliff’s δ values shown for *p* < 0.1. CRB = cerebrolysin; rTMS = repetitive transcranial magnetic stimulation; PLC = placebo; n.s. = not significant; • - *p* < 0.1: * - *p* < 0.05; ** - *p* < 0.01; *** - *p* < 0.001.

The parietal region also showed differences between visits in the delta *(p* = 0.03) and alpha (*p* = 0.012), but not theta (*p* = 0.777) and beta (*p* = 0.204) power spectral densities between visit, with no significant differences between the treatment groups (Figure 4). Specifically, there was a medium difference between the visits regarding the delta power in the CRB + sham group (*p* = 0.005, *δ* = 0.467).

**Figure 5.**
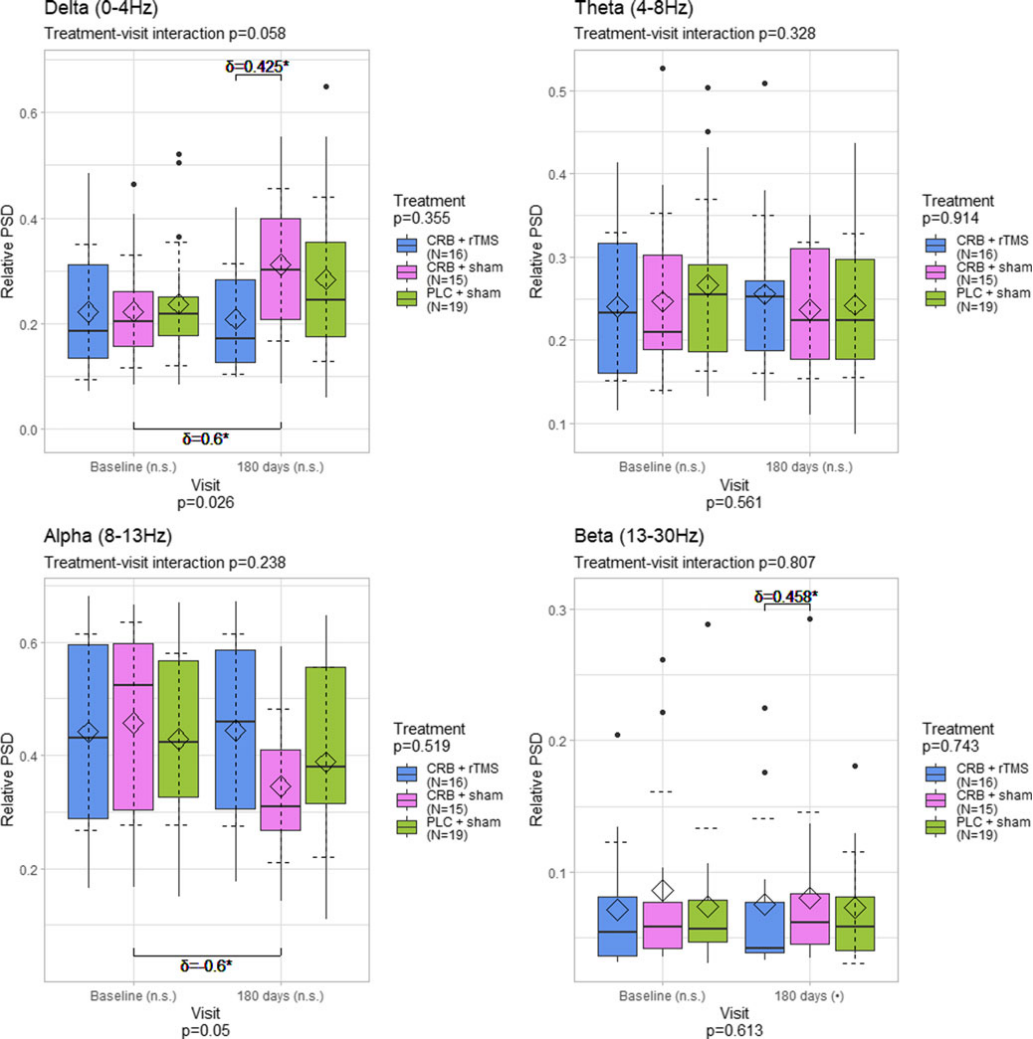
Distribution of power spectral density values averaged for the frontal electrodes, by frequency band, visit, and treatment group. The diamond represents the mean, and the dashed error lines represent the standard deviation relative to the mean. Resampling-based mixed-models ANOVA p-values specified for each factor; Kruskal–Wallis test was used for comparisons between all groups for each visit; Wilcoxon rank-sum test and Cliff’s δ were used for comparisons between groups (upper part of the graphs); Wilcoxon signed rank test and paired Cliff’s δ were used for comparisons between visits (lower part of the graphs); Cliff’s δ values shown for *p* < 0.1. CRB = cerebrolysin; rTMS = repetitive transcranial magnetic stimulation; PLC = placebo; n.s. = not significant; • - *p* < 0.1: * - *p* < 0.05; ** - *p* < 0.01; *** - *p* < 0.001.

The frontal region showed the same changes (delta *p* = 0.026, alpha *p* = 0.05), but further analysis generally revealed smaller differences, with only medium-sized increases in delta power (*p* = 0.018, *δ* = 0.6) and decreases in alpha power (*p* = 0.048, *δ* = 0.6) for the CRB + sham group (Figure 5).

Lastly, occipital and temporal regions showed the same results, with lower effect sizes.

**Figure 6.**
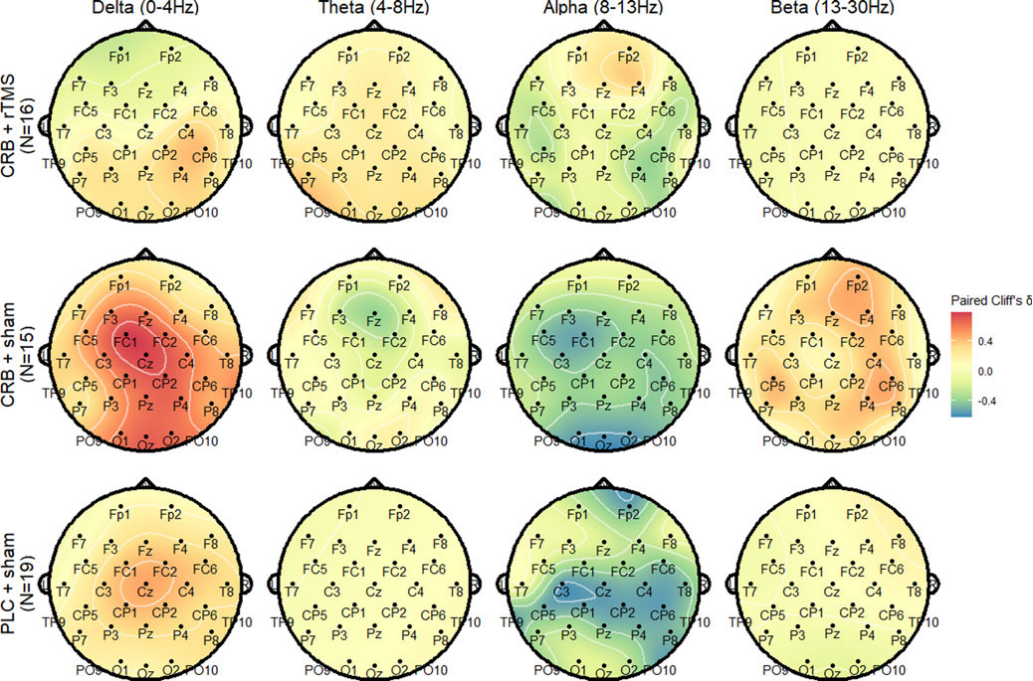
Topoplot representation of changes in relative power spectral density compared to baseline levels, by group and frequency band. For paired Cliff’s δ, negative values (green and blue) show decreases and positive values (orange and red) show increases.

Further analysis of individual electrodes shows that the most prominent changes in relative power spectral density appear in the group treated with CRB only, in the fronto-central region, in median and left electrodes (Figure 6).

Detailed results, by scalp zone and frequency band, as well as 2D representation of the position of electrodes for each scalp zone, are available in Supplementary File S2.

### Correlation of power spectral density values with neuropsychological metrics

**Figure 7.**
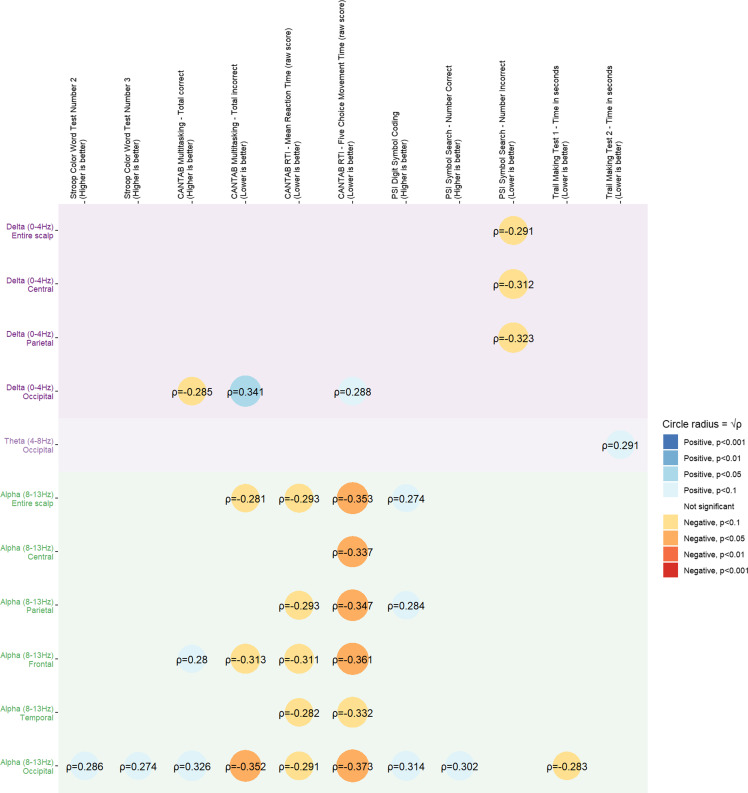
Correlation matrix between neuropsychological metrics and power spectral density values, using Spearman’s correlation coefficient (*ρ*) and its associated statistical test, and based on values from all groups and both visits. MoCA = montreal cognitive assessment; CANTAB = Cambridge neuropsychological test automated battery; OTS = one touch stockings; PSI = processing speed index; RTI = reaction time. Data was available from all 50 patients for non-CANTAB tests (resulting in 100 pairs of values for each correlation done on visit data), and for CANTAB tests, 97 pairs of values were available, due to two patients not having valid data for the first visit, and one patient not having valid data for the third visit; p-values were adjusted using the false discovery rate method; the matrix was collapsed showing only rows (frequency band and zones) and columns (neuropsychological tests) with at least a correlation with adjusted *p* < 0.1.

We analyzed the correlations between neuropsychological metrics and resting-state power spectral densities. The analysis done on combined raw data from both visits and all three groups revealed weak to moderate correlations between alpha-band resting-state power and the different tests applied, with higher cognitive performance being associated with stronger alpha activity; moreover, there were also weak correlations between delta-band resting-state power and some tests (Figure 7). Overall, the occipital region had the highest correlations with test results, and specifically, only the occipital region alpha-band power spectral density was significantly correlated with Stroop Color Word tests results (occipital region *ρ* = 0.191, *ρ* = 0.286 and *ρ* = 0.274 compared to entire scalp *ρ* = 0.126, *ρ* = 0.187 and *ρ* = 0.181, for Tests 1, 2 and 3 respectively). For CANTAB, lower reaction times and multitasking performance correlated with alpha activity, especially in the frontal (*ρ* = −0.361 and *ρ* = −0.313 respectively) and occipital (*ρ* = −0.373 and *ρ* = −0.352) zone, compared to the general activity (*ρ* = −0.353 and *ρ* = −0.281).

## Discussion

The present study represents a secondary data analysis of the CAPTAIN-rTMS randomized-control trial; while the original study aimed to investigate the cumulative efficacy of CRB + rTMS compared to CRB monotherapy (through randomly allocating patients to the double intervention - CRB + rTMS, single-therapy - CRB + sham, or no-intervention group - PLC + sham), this secondary analysis aimed to characterize the changes in resting-state power spectral density profiles on quantitative electroencephalography for patient with TBI receiving each of the proposed therapies, further correlating the relative PSD values with . neuropsychological measures.

The comparison of baseline EEG recordings and 80 days after treatment completion revealed mostly nonsignificant statistical changes for CRB + rTMS and PLC + sham groups. However, in the CRB monotherapy group, an increase in delta power and a concurrent decrease in alpha power were observed post-intervention. This is in line with our previous results, that delta/alpha ratio and (delta+theta)/(alpha+beta) ratio of the entire scalp and of the frontal zone were increased in the group treated with CRB only, at the last visit compared to the first, while no such effect was seen for theta/beta ratio nor for other treatment groups [19]. Still, while the results of the previous study have the advantage of summarizing the overall tendencies of electroneurophysiological activity through a reduced number of values (three ratios), studying the four proposed frequency bands separately and through different scalp zones provides a better view of the changes suffered by each treatment group individually. Namely, the proposed ratios have the disadvantage of not being able to discriminate between delta and theta changes, respectively between alpha and beta, but only between slow and fast activity. Moreover, the current analysis provided information regarding the associations of such activity profiles with neuropsychological performance. Taking into consideration the availability of classical EEG equipment and of open-source software for qEEG computation, we believe further research in this area could pave the way towards using quantitative electroencephalography as a marker of cognitive recovery or decline in the medical practice.

These findings are inconsistent with the majority of EEG studies examining the effects of CRB treatment on brain activity [40–43]. For instance, an exploratory clinical trial conducted by Alvarez and colleagues reported chronic neuroplastic effects as demonstrated by decreases in slow-frequency activity accompanied by enhancement of fast brain activity after CRB administration in post-acute TBI patients [40]. Previous studies performed by our team indicated dose-dependent effects on induced long-term changes on brain activity, with higher doses of CRB (30 mL doses five days/week for four weeks) being more efficacious than lower doses (10 mL) in reversing the EEG slowing pattern in the treatment of vascular dementia [42, 43]. Furthermore, the observed changes in brain activity within this group may be accompanied by poorer rehabilitation, given that alpha power serves as a critical qEEG parameter for predicting long-term outcomes [44]. Different from our study, patients of Alvarez and colleagues were already being treated with stable doses of neuroprotective compounds such as citicoline and piracetam by the time CRB was administered [40, 41]. Indeed, integrating multi-modal treatment approaches targeting different secondary insults has demonstrated acknowledged superiority in both clinical and preclinical studies [45].

The presence of higher levels of delta power after an acute event (e.g., TBI) is a well-known change of the spectral profile [46–48]. The work of Franke et al. [49] reports its occurrence in TBI patients where rTMS or sham stimulation was applied, with no other means of treatment incorporated (e.g., neuroprotective agents). In our work, the CRB + sham group exhibited relevant delta power elevations after 180 days, of a larger magnitude than in the CRB + rTMS group and close to the ones found in the sham+placebo group. It is therefore difficult to consider if the respective changes are influenced by CRB itself, are the result of the natural evolution of TBI, or multimodal approaches are needed. For a pertinent conclusion, we consider that three premises should be considered. Firstly, the reports of previous publications, describing large amounts of low band activity in the recordings of TBI patients, either through qualitative (e.g., visual inspection) or quantitative (e.g., spectral analysis) means, even in situations where no active compound (including CRB) was used [48, 49]. Secondly, the possible beneficial role of CRB in the reduction of delta activity following TBI, found in some related papers [40, 41]. And thirdly, while the CRB + sham regime indeed displayed higher delta power during the second assessment, the CRB + rTMS one led to lower increases (central) or even mild decreases (whole scalp, parietal, frontal) of delta activity when looking at the mean value (Figures 2–5). Taking all premises together, we incline that although CRB itself has been found to mitigate the normalization of the EEG profile in TBI patients, moderate or moderate-to-severe TBI cases could necessitate intensive treatment regimes, like ones involving two different modalities (e.g., CRB + rTMS).

Patients who suffered TBI six years prior and had an eyes-closed EEG analysis, have shown to have lower overall absolute alpha power, but also decreased theta and delta absolute powers in caudal (posterior) regions; patients who had higher neuropsychological impairments also had lower spectral powers, compared to patients with mild or no deficit [50]. The main difference between that study and ours is the employment of relative power computation, which better represents the profile of the cerebral activity and is not as influenced by the physical contact between the electrode and the scalp.

Patients with spinal cord injury and chronic pain also had lower relative alpha power, both compared to spinal cord injury patients that did not accuse pain, as well as compared to healthy controls, although the authors propose mechanisms based on thalamocortical dysrhythmia as a secondary effect of spinal cord injury, as well as related to sleep pattern disruption by pain [51].

He et al., 2020 [52] developed a predictor algorithm to differentiate patients with unresponsive wakefulness syndrome (mixed etiology, stroke and TBI) with high likelihood of clinical improvement after 4-weeks of left dlPFC rTMS treatment, based on the ratio of alpha power before intervention at the level of superior parietal cortex (P3 electrode) and delta power after rTMS treatment in the left DLPFC (F3 electrode). Therefore, this electrophysiological discriminator between responders/non-responders after rTMS, identifies in this scenario the chance of clinical recovery, respectively to regain consciousness.

Few EEG studies have examined the effect of different stimulation locations on clinical progression after brain injury. Evidence from a double-blind, randomized-control trial, suggests that high-frequency rTMS (20 Hz) over the primary motor area M1 of the affected hemisphere determines a superior therapeutic outcome (assessed behaviorally, with EEG standard grading and somatosensory evoked potentials of the upper limb) as compared with left DLPFC or placebo in severe TBI patients with unresponsive wakefulness syndrome [53]. Recently, a positive clinical effect induced by 10 Hz rTMS stimulation over the parietal cortex (precuneus, Pz electrode), accompanied by markers of cognitive updating (P300 wave amplitude) were registered in patients with severe brain injury [54]. Although most studies investigated the clinical spectrum more closely, there`s a general lack of agreement regarding which stimulation target benefits different etiologies, disease severities and lesion locations. An ongoing randomized-controlled, crossover study aims to determine the optimal stimulation site based on individual behavioral and electrophysiological efficacy [55].

This secondary analysis has several limitations related to the design of the original study. First, the sample size was lower than that of the original study. This was because of the lockdown restrictions during COVID-19 pandemic, which prevented us from recording EEG“s of all patients. Moreover, we performed multiple comparisons, especially across the three treated groups, which would have increased the risk of Type I errors; we partially accounted for this limitation through adjusting the p-value using the False Discovery Rate method. Related to the qEEG computation, we analyzed eyes-closed segments of the recording; EEG recordings during other tasks, especially during cognitive tests, had many artifacts, which would have prevented further analysis. Cerebrolysin was administered out of its therapeutic window [56], which was done both due to the possible epileptogenic effects of CRB, as well as due to the increased risk of posttraumatic epilepsy [3]. Lastly, no information regarding TBI sites or medical history of the patients was available; a granular description of TBI sites would result in multiple subgroups available for analysis, which combined with the fact that this was a small-scale study would result in statistical results with low power. While future studies could address the lack of information regarding TBI sites, it would be necessary for such studies to have higher sample sizes.

Several factors need to be taken into consideration in future studies. Both rTMS-dependent parameters such as stimulation frequency, dosing, train length, session number, as well as CRB treatment scheduling, necessitate further calibration. Concurrent medications that might influence EEG activity, clinical scores, and the differences registered between treatment groups should be systematically analyzed and controlled. Lastly, further analysis of qEEG indices, both in eyes-closed segments as well as during cognitive tasks, is warranted.

Regarding CRB, recent clinical trials researched either its late administration in progressive neurological disorders (such as amyotrophic lateral sclerosis [57], cervical spondylotic myelopathy [58, 59], anosmia and ageusia after COVID-19 [60] or even in preterm infants [61] or cerebral palsy [62]), or its early administration in sudden onset neurological diseases (such as TBI, ischemic stroke [63–66] or aneurysmal subarachnoid hemorrhage [67]). In ischemic stroke, CRB is recommended for early motor neurorehabilitation by European Academy of Neurology and European Federation of Neurorehabilitation [56]. In the current study, the effect of CRB might be dependent both on time from disease onset, as well as disease severity: in a study regarding ischemic stroke for example, CRB was effective in patients with severe motor involvement, but not when analyzing all the patients [64]; CRB (alone or combined with donepezil) also showed a positive effect on the levels of some neuronal-derived extracellular vesicles which were used as biomarkers of Alzheimer’s disease severity [68]. In other cases, while CRB was deemed safe, it did not significantly aid in the functional recovery of the patient, such as in aneurysmal subarachnoid hemorrhages [67]. Thus, further research regarding the late administration of CRB in sudden onset neurological diseases is warranted.

Regarding rTMS protocols for TBI, there is high variability especially regarding the instrument used, number of sessions (days), pulse trains length and intervals [69], as well as in the targeted disease, some studies aiming to solve complications associated with TBI such as depression [70, 71], cognitive disability [72]. Repetitive transcranial magnetic stimulation was also used by Shen et al. [73] in post-TBI vegetative state patients, where results showed improvements in Glasgow Coma Scale scores, Coma Recovery Scale scores, as well as in EEG and somatosensory evoked potentials activity; stimulation of the left motor cortex proved to be the most effective in recovery of such patients, but left DLPFC stimulation also proved more effective than sham stimulation. Leung et al. [74] used rTMS on the left motor cortex to treat post-TBI headache. Horton et al. [75]. targeted the auditory cortex in order to treat post-TBI tinnitus. Other groups used imagistic or neuroelectrophysiologic approaches to identify candidate areas for transcranial magnetic stimulation for each patient individually [76]. Repetitive transcranial magnetic stimulation of right DLPFC was successfully used as a precursor for amantadine in treating disorders of consciousness in post-TBI patients [77]. Thus, future research should better define the optimal rTMS protocol for treating TBI and its complications.

In conclusion, we found a slowing of brain waves during the recovery after TBI, especially in the central and parietal regions; those differences were further influenced by dosing regimens of both CRB and left dorsolateral prefrontal cortex rTMS. Resting-state relative power spectral densities correlate with neuropsychological measurements, with relative alpha power being associated with higher performance and relative delta power being associated with lower performance.

## Supporting information

10.1017/cts.2025.10159.sm001 Livinț-Popa et al. supplementary material 1 Livinț-Popa et al. supplementary material

10.1017/cts.2025.10159.sm002 Livinț-Popa et al. supplementary material 2 Livinț-Popa et al. supplementary material

10.1017/cts.2025.10159.sm003 Livinț-Popa et al. supplementary material 3 Livinț-Popa et al. supplementary material

## Supplementary material

The supplementary material for this article can be found at https://doi.org/10.1017/cts.2025.10159.
