# Supplementary figures and images for: Delta power surge and alpha power decline in traumatic brain injury recovery: A quantitative EEG analysis of the CAPTAIN-rTMS trial
Source: J Clin Transl Sci. 2025 Sep 25;9(1):e236. doi: 10.1017/cts.2025.10159 (PMC12695505; doi:10.1017/cts.2025.10159)

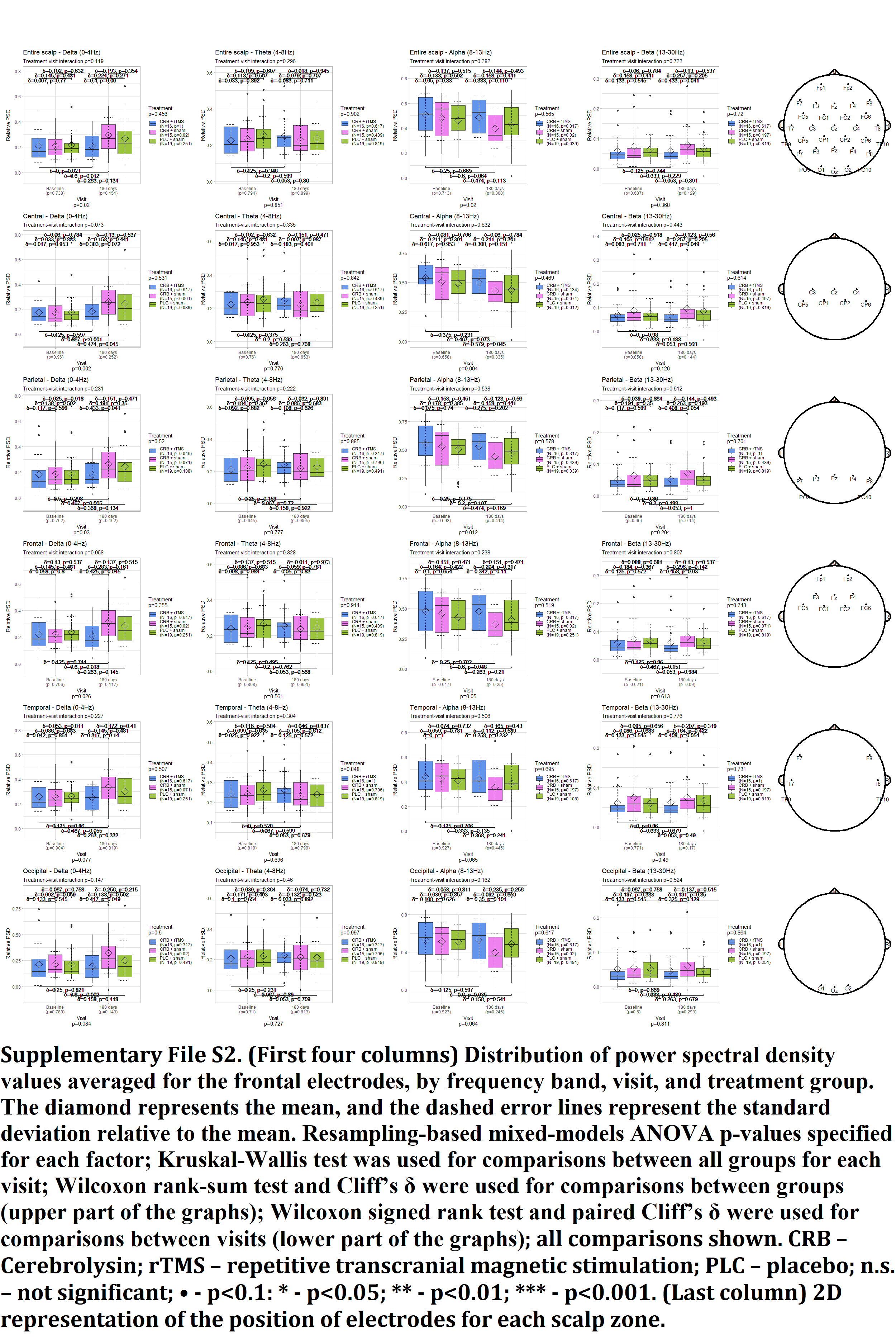

Supplement: Livinț-Popa et al. supplementary material 2 — Livinț-Popa et al. supplementary material [file S2059866125101593sup002.zip › S2_In-depth relative PSD results_uploaded.png]

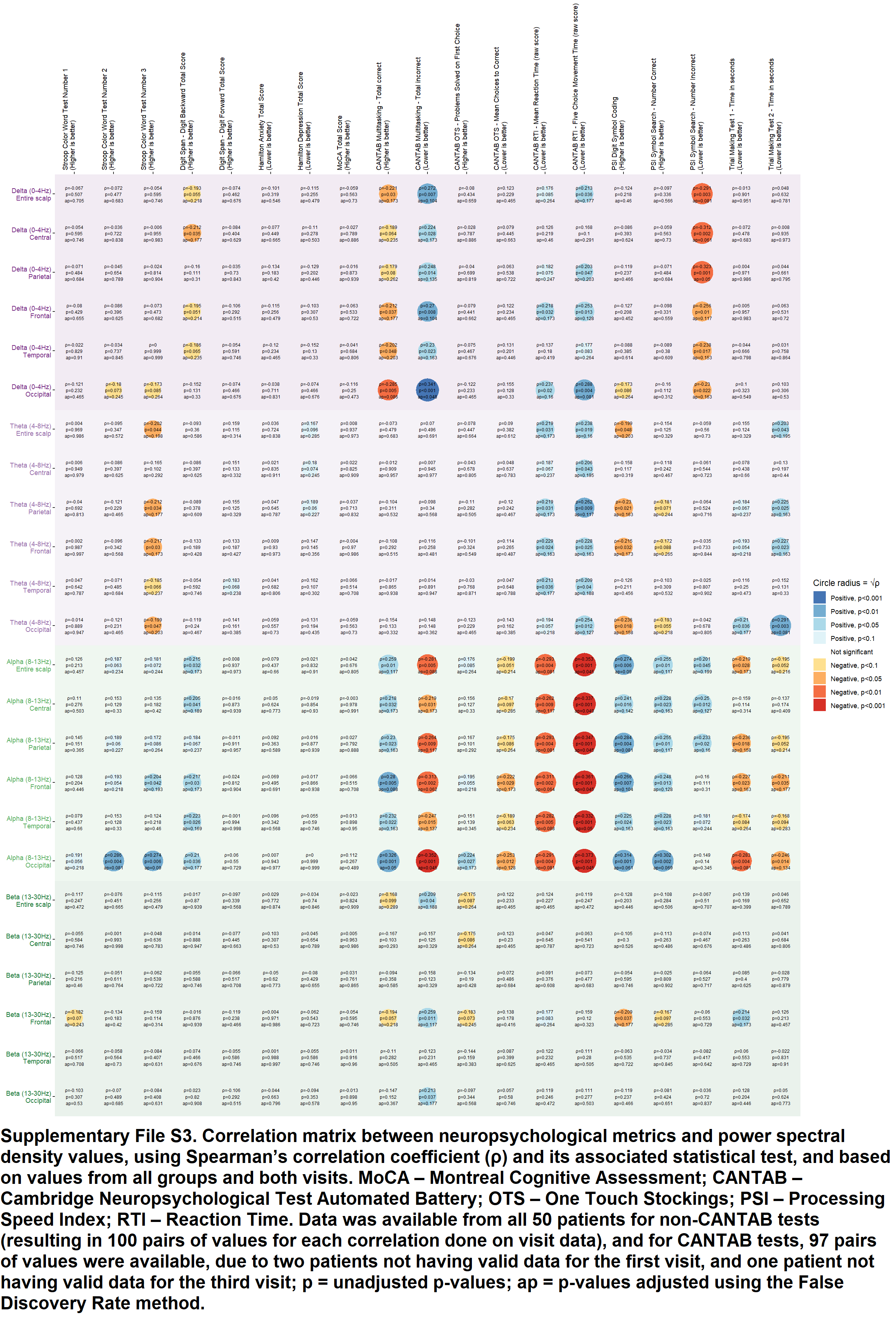

Supplement: Livinț-Popa et al. supplementary material 3 — Livinț-Popa et al. supplementary material [file S2059866125101593sup003.zip › S3_Correlation Matrix_uploaded.png]
